# Engagement with a diverse Stakeholder Advisory Council for research in dementia care

**DOI:** 10.1186/s40900-021-00297-8

**Published:** 2021-07-23

**Authors:** Sara S. Masoud, Ashlie A. Glassner, Neela Patel, Mayra Mendoza, Deborah James, Sheran Rivette, Carole L. White

**Affiliations:** 1 The University of Texas Health Science Center at San Antonio, 7703 Floyd Curl Dr, San Antonio, TX 78229 USA; 2 The Glenn Biggs Institute for Alzheimer’s and Neurodegenerative Diseases, University of Texas Health Sciences Center, 7703 Floyd Curl Dr, San Antonio, TX 78229 USA

**Keywords:** Engagement, Stakeholders, Dementia, Caregivers, Research, Council

## Abstract

**Background:**

The inclusion of stakeholders throughout the research process has been gaining recognition as an approach that can improve the quality and impact of research. Stakeholder engagement for dementia care research has been identified as a national priority, though evaluation of engagement strategies and their impact has been limited. In dementia care research, stakeholders can include individuals living with dementia, family care partners, and health and social care professionals in dementia care. A Stakeholder Advisory Council (SAC) was established to identify priorities for dementia care research that are most important to stakeholders. Strategies to build capacity for research and facilitate engagement among the SAC were used to identify the research priorities. This study describes the experiences of SAC members engaged in the research process.

**Methods:**

To evaluate stakeholder engagement, semi-structured interviews were conducted with members of the SAC to understand their experiences and perspectives on the strategies used to facilitate engagement and build capacity for research. Interviews were recorded, transcribed, and thematically analyzed using a mixed inductive and deductive approach. Findings were presented to members of the SAC to determine whether they felt their perspectives and experiences were accurately represented. Final domains and themes presented here were approved by the SAC.

**Results:**

Interviews (*N* = 11) were conducted with members of the SAC representing each stakeholder group; persons living with dementia (*n* = 2); family care partners (*n* = 4), and health and social care professionals in dementia care (*n* = 5). Ten themes were categorized into four overarching domains: accessibility, council infrastructure, values and environment, and benefits of involvement.

**Conclusions:**

Findings from this qualitative study are a resource for researchers seeking to collaborate with diverse stakeholder groups to represent their perspectives in research, including individuals living with dementia. The domains and themes identified here support the inclusion of diverse stakeholders in the research process, centering engagement and capacity building strategies around individuals living with dementia.

**Supplementary Information:**

The online version contains supplementary material available at 10.1186/s40900-021-00297-8.

## Plain English summary

Involving stakeholders in research about dementia care is recognized as an important approach to develop findings that are relevant to individuals living with dementia, family care partners, and health and social professionals in dementia care. Still, this approach is not often used and there is a need for more information about how researchers can engage stakeholders in the research process and the impact of engagement from the perspectives of stakeholders. In this paper, we describe how a mixed group of stakeholders was engaged for a project to identify priorities for dementia care research and their perspectives of the experience.

A Stakeholder Advisory Council (SAC) worked together to develop their skills in research and connected with their communities to determine what is most important to be studied in the field of dementia care research. To understand the perspectives of the stakeholders involved in this project, we conducted individual interviews with members of the SAC.

This study will help researchers understand how they can collaborate with stakeholders in dementia care and the benefits of including individuals living with dementia, family care partners, and professional stakeholders in the research process.

**Supplementary Information:**

The online version contains supplementary material available at 10.1186/s40900-021-00297-8.

## Background

The inclusion of stakeholders in the development, implementation, and dissemination of research has been gaining importance among researchers. There is  a growing recognition of how stakeholder engagement can contribute to the quality of research, addressing topics that are most meaningful to patients and the public [1]. Stakeholders in this context are defined as individuals or groups who are affected by the conduct and the results of health-related research [2]. Funding agencies are helping to advance this philosophical and methodological shift in how research is conducted, with many agencies, including the Patient Centered Outcomes Research Institute (PCORI) in the United States and INVOLVE, funded by the National Institute of Health Research in the United Kingdom, supporting the inclusion of stakeholders in research [3, 4].

Community engaged research is an increasingly utilized practice to develop research that is translatable and representative of the perspectives of the community of interest [5, 6]. Authentic engagement of stakeholders in research means that stakeholders actively participate in activities that were traditionally within the domain of researchers, collaboratively setting research priorities and serving in roles that may include co-investigator, helping to design a research project, collecting data, interpreting the data, and disseminating results [1, 7]. Stakeholder collaboration challenges the traditional views of the expert versus the lay person and recognizes the value of both ‘expert by training’ and ‘expert by experience’. [5].

While patient and public involvement in research is becoming more common [8, 9], persons living with dementia and their care partners continue to face barriers and inadequate opportunities to participate as research partners [10, 11]. Dementia affects almost 50 million people worldwide and is predicted to triple by 2050 [12, 13]. The projected increase poses significant economic, healthcare, and social threats worldwide. The growing numbers of individuals and families impacted by dementia underscores the importance of engaging these stakeholders in research, generating questions and conducting research that is relevant and meaningful to them. Researchers may hold diverging perspectives from stakeholders about what is most important to study in dementia care [14–17]. Researchers and stakeholders often have mismatched priorities related to health research, often reflecting divergent views around preferences for pharmacological or non-pharmacological disease management and treatment [2, 18]. A recommendation from the Research Summit on Dementia Care, Services, and Supports to address potential mismatches in priority setting for dementia care research was the engagement of persons living with dementia and their care partners in research teams [19].

Community and stakeholder advisory councils, often referred to as Community Advisory Boards (CABs), have been established as an effective strategy to develop research that is responsive and relevant to the end-users [20, 21]. These councils are typically comprised of representatives of stakeholder groups that reflect the community of interest such as a shared experience related to an illness, identity markers, roles, or cultures, among others [22]. Community and stakeholder advisory councils often are established to inform research, where community members are able to voice their perspectives and priorities related to health concerns in the academic and clinical research spaces from which they are traditionally excluded [23]. Members of these councils are considered partners to research teams, engaged at every step of the research process to lend their perspectives and expertise in identifying priority issues, designing studies, implementing a study or project, and disseminating knowledge and findings [24].

The infrastructure of a stakeholder advisory council may be an effective approach to engage persons living with dementia and family care partners in research alongside health and social care professionals. Stakeholder advisory councils that include family care partners have been shown to be effective in informing the development and implementation of interventions to support family care partners of persons living with dementia [25]. Bethell et al. conducted a scoping review, describing patient and care partner engagement in dementia research [17]. While family care partners, health professionals, and researchers have been engaged in stakeholder advisory council settings, persons living with dementia have had fewer opportunities to participate in research and program development in a similar capacity. Exclusion from these settings may be associated with challenges related to the progressive nature of the condition, potential to overburden families who are already experiencing increased responsibilities and challenges related to a diagnosis, and concerns over the feasibility of having persons living with dementia as co-researchers [26]. One potential approach that may support representation of persons living with dementia as collaborators in research is the establishment of mixed advisory councils that include other constituents of diverse stakeholder groups alongside those living with dementia. Stakeholder advisory councils comprised of diverse representative groups who have a vested interest in dementia research may be compelling to those who aim to reflect the priorities of a broad group of constituents who are all potentially impacted by the results of the research.

While there is evidence suggesting that stakeholder engagement is key in developing and implementing research, there is limited empirical evidence on the best practices for stakeholder engagement [27]. There are few studies that have examined the impact of participation of persons living with dementia and their care partners on the research process [17, 28]. To address this gap, our purpose is to describe the experiences of members of a stakeholder advisory council (SAC) who were engaged for a project to identify and prioritize topics for patient centered outcomes research (PCOR) in dementia care. The SAC were engaged in capacity building activities throughout the project period to support them in providing guidance and oversight of the project timeline and deliverables. The SAC included persons living with dementia and care partners alongside health and social care professionals in dementia care who partnered with the project team to build capacity for PCOR and evaluate the engagement process.

## Methods

### Establishing and engaging a diverse stakeholder advisory council

To build capacity for dementia care research and identify research priorities from a multi-stakeholder perspective, the project team invited people impacted by dementia personally or professionally to participate in a SAC. Specifically, the SAC was formed to identify gaps in dementia care from multiple stakeholder perspectives and, working with their community networks, developed a prioritized list of topics for patient centered outcomes research (PCOR) to address the gaps. Findings from the prioritization of stakeholder-driven topics for dementia care PCOR are described elsewhere [15]. Members of the core project team were well positioned to invite stakeholders to participate on the SAC as they included a faculty researcher in caregiving who is director of a well-established community program to support families living with dementia, providers of palliative care including a geriatric clinician researcher and nurse faculty, and a caregiver specialist who had cared for a partner living with dementia and since applied her expertise to support other families living with dementia. Stakeholders for this project were identified as anyone who may be directly impacted by research in dementia care, including persons living with dementia and their care partners, palliative care clinicians and nurses, organizations supporting families living with dementia, and other social support professionals including pastoral care providers and care management programs.

A goal of the project team was to include perspectives of different representative groups while centering around the experiences and needs of the ultimate end-users of dementia care, those living with dementia and those caring for individuals living with dementia. Diversity among the SAC is primarily attributed to their roles as individuals representing different professions and lived experiences related to dementia. The SAC included 2 persons living with dementia, 4 care partners, 7 health and social care professionals, and 2 researchers (Table 1). SAC members also represented different age groups, some caregivers were adult children who were currently providing or had provided care for a parent(s) living with dementia. Other caregivers were older adults who had cared for a spouse living with dementia in the past or were currently caring for their spouse living with dementia who were also members of the SAC.

**Table 1:** Stakeholder groups represented on the council

**Persons living with dementia**	2
Alzheimer’s disease (*n* = 1)	
Lewy Body (*n* = 1)	
**Family care partners**	4
Adult children (*n* = 1)	
Spousal (*n* = 3)	
**Health and social care professionals**	7
Geriatric and Palliative Care Physician (*n* = 2)	
Palliative Care Nurse (*n* = 1)	
Hospice Chaplain (*n* = 1)	
Community Organizations (*n* = 2)	
Memory Care (*n* = 1)	
**Researchers**	2
Research lead (*n* = 1)	
Research coordinator (*n* = 1)	

The SAC met monthly over a two-year period. In the first year the SAC was chaired by the project lead and in the second year, was co-chaired by a person living with dementia who partnered with the project lead to set meeting agendas, facilitate discussions, and plan project deliverables. Initial meetings were held at a university then relocated to the offices of the local Alzheimer’s Association chapter in order to invite wider community groups to join the SAC for discussions on research priorities. Three meetings took place at the new location before they were ultimately transitioned to a virtual format following social distancing limitations due to COVID-19. Meetings typically followed a 3–5 item agenda, although open dialogue was always encouraged and co-chairs were prepared to adapt meetings to address issues pertinent to the SAC. Agenda items were informed by project deliverables and the interests of the SAC related to their building capacity goals for engagement in PCOR (i.e., review of peer-reviewed publications). The co-chairs of the SAC, a person living with dementia and the project lead (academic researcher), would meet prior to each SAC meeting to discuss the timeline of the project and decide on agenda items. At every SAC meeting, an open agenda item created space for SAC members to recommend upcoming agenda items or to discuss topics or concerns not included on the agenda for that day. Detailed notes were taken at every monthly SAC meeting which were e-mailed to the members and archived in an online community. Meetings held virtually were recorded, uploaded to a private online platform, and made accessible to members of the SAC.

Building capacity for research was critical to mobilize the SAC to connect on behalf of the project with their wider networks of stakeholders (Table 2). Within the first 6 months of the project, the *Core Principles for Involving People with Dementia in Research* by the Scottish Dementia Work Group for Research were shared, reviewed, and discussed in-depth at multiple SAC meetings to reinforce that respect and support for persons living with dementia was at the forefront of the group’s collaborative efforts [29]. Webinars were planned by the SAC based on their interests and identified needs regarding education and awareness in areas relevant to research. A member of the Institutional Review Board (IRB) attended a meeting to facilitate discussion around research practice and ethics. Researchers periodically summarized dementia care studies in presentations by adapting the information from peer-reviewed articles into more accessible language with visualizations. The group could then engage in conversations about the studies and make relevant connections. Annual symposia were hosted by the SAC to support capacity building efforts for engagement in research and to include their community networks in learning about their progress in the project. Annual symposia were adapted to the virtual context following COVID-19 in collaboration with the SAC.

**Table 2:** Research capacity building activities of the SAC

Project Years	2018–2019				2019–2020			
Calendar Quarters	Q1	Q2	Q3	Q4	Q1	Q2	Q3	Q4
IRB workshop session	x							
Reviewed & discussed *Core Principles for Involving People with Dementia in Research*		x						
Facilitated article reviews and discussions^a^	x		x		x			
Webinars about PCOR and dementia care		x		x				
Annual research symposiums			x				x	
Survey data collection methods workshop						x		
Survey review and feedback							x	
PCORI Annual Symposiums: Attendance and group debrief			x				x	
Alzheimer’s Disease International Conference: Attendance and group debrief								x

^a^Article reviews and discussions were facilitated at multiple SAC meetings during quarterly periods

Prior to the COVID-19 pandemic, the project team assisted a member of the SAC to obtain a scholarship to attend the PCORI Annual Conference in Washington, DC. This member later summarized notable presentations from the conference for the rest of the SAC. They were also directed to the PCORI website where they could access all conference materials and recorded presentations at no cost. In December 2020, SAC members were registered for the Alzheimer’s Disease International conference at which they could participate in numerous virtual scientific presentations and discussions.

### Evaluation

To evaluate stakeholder engagement, semi-structured interviews with members of the SAC were conducted to understand their experiences as council members and their perspectives on the strategies used to facilitate engagement and build capacity for research. The interviews sought to understand diverse stakeholder perspectives on the approach to engagement centered around the experiences of SAC members living with dementia. To do this, an interview guide was developed using the *Core Principles for Involving People with Dementia in Research* as a framework [29]. The *Core Principles* include: (i) how people with dementia are valued and involved in research, (ii) lived experience as valid knowledge, (iii) physical and emotional safety, (iv) accessibility of all aspects of research, (v) training for researchers and (vi) the impact of our experiences of time on research processes [29]. The interview guide was revised and reviewed by the study team. Questions were designed to be somewhat broad while also addressing each of the *Core Principles*. After completing the first interview with a care partner, some questions were adapted and reformatted to ensure they were accessible for all council members, including those living with dementia.

To limit respondent bias, interviews were conducted by two research assistants who had not been closely involved in the project. At the time when interviews began, the COVID-19 pandemic had limited the ability to conduct any in-person activities. As such, all interviews were coordinated via email and phone and were conducted using Zoom videoconferencing software, except for one interview which was conducted by telephone. Participants were provided a detailed information sheet which was read aloud by the research coordinator upon invitation and repeated prior to beginning the interviews. Upon receiving permission from the participants, all interviews were recorded and later transcribed. To ensure that persons living with dementia were comfortable to freely share their perspectives, they were given the option to participate in interviews with or without their care partner present, and if present, the care partner was asked prior to the interview to refrain from speaking on behalf of their partner living with dementia and instead to serve in a supportive role. In addition to exploring the six areas of engagement outlined by the *Core Principles*, participants were invited to speak to the potential benefits and challenges they faced as a member of the SAC. Participants were also asked to reflect on how their work on the council would contribute to dementia care research and their involvement in a research project.

This project was submitted to the IRB prior to beginning the interviews and determined to be non-regulated research (HSC20200470N). We have included the Guidance for Reporting Involvement of Patients and the Public (GRIPP2 Short Form) checklist in Supplementary Materials [30].

#### Analysis

Transcribed interviews were analyzed independently by two members of the research team using a mixed inductive and deductive thematic analysis approach. The *Core Principles* were used as a sensitizing framework, with new codes and themes identified from the interviews. After initial coding of all transcripts, the two coders reviewed and compared their themes and domains. Although minimal reconciliation was needed, the lead researcher was consulted for any discrepancies between coders. The results were presented to the SAC at two monthly meetings to invite their input. Members of the SAC were encouraged to share any additional insights and to determine whether they felt the themes accurately reflected their perspectives and experiences. The final domains and themes were approved by the SAC who confirmed their perspectives as a group were accurately and respectfully represented. This evaluation is co-authored with two family care partners from the SAC (MM, SR) who reviewed the findings, provided feedback for the analysis, revised drafts of the manuscript, and approved the final draft before submission. Their insight as care partners and members of the SAC who also bring valuable knowledge and skills beyond their care partner roles ensures that the perspectives of key stakeholders are engaged throughout the process and in the final representation of the engagement experience.

## Results

Eleven interviews were conducted with respondents representing each stakeholder group on the SAC; persons living with dementia (*n* = 2), family care partners (*n* = 4), and health and social care professionals (*n* = 5). In the “health and social care professionals” category a geriatric and palliative care physician, palliative care nurse, hospice chaplain, and two community organization staff members were interviewed. The project lead and key personnel who were closely involved in the planning, coordination, and facilitation of the project’s research capacity building and engagement activities were excluded from interviews. Other SAC members who were not interviewed were excluded due to scheduling challenges, though final respondents did include representatives of each of the various stakeholder groups on the council.

**Fig. 1:**
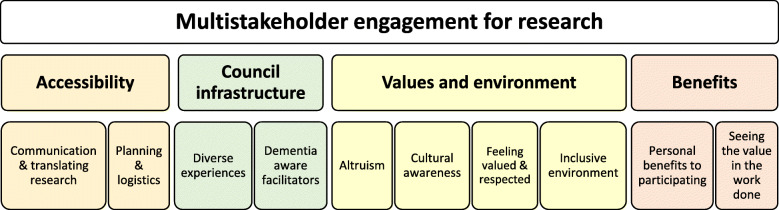
Domains and themes reflecting key principles for engaging diverse stakeholders in dementia care research

Key principles important for successful multi-stakeholder engagement that center around persons living with dementia were identified. These principles are categorized into four overarching domains: accessibility; council infrastructure; values and environment; and benefits of involvement. These domains and themes are presented visually in Fig. 1.

### Accessibility

It was important to SAC members that the group used a common language to communicate and translate research in an accessible and inclusive way. This was reflected in their preference to have scientific literature adapted by the project team into more accessible forms of information delivery, particularly by way of visual alternatives. Peer-reviewed publications were printed in enlarged fonts for the SAC given at least 1 month prior to the facilitated discussions of the literature during meetings, although reading beforehand was optional. During discussions of scientific literature, the project team would select pertinent excerpt and adapt into PowerPoint slides, providing printed copies of the slides and original publication available to SAC members for reference and notetaking if needed. Respondents reflected that not only did they prefer when information was shared in an accessible structure and format, but that they appreciated when the SAC and project team would avoid the use of technical jargon and would deliver information, revisiting and reinforcing the information repeatedly over the project period during meetings and symposia.

*“[The project lead] was very cognizant that not everybody in the room had a large or small research background. We started everything at the beginning…….Visuals. Visuals. Visuals, visuals, visuals*.” – Family care partner 2

*“…[the project team] deliver it in such a synchronized way that if you didn't get it the first time, you're gonna get it the second time. If you didn't get it last week, then you're gonna get it at this meeting*.” – Health and social care professional 4

*“They’ll go back and talk through it again and that helps.”* – Person living with dementia 1

How the project team approached the planning and logistics of engagement and research capacity building activities was a key subtheme reflecting how SAC members perceived the accessibility of the project. The meeting times and locations were important to all members of the SAC. The community members’ needs were prioritized when choosing meeting times and locations, although the logistics were challenging to ensure all SAC members could participate in every meeting. Clinicians were challenged by busy clinic schedules while community members were challenged to commute to the traffic-dense university setting. The meeting times and locations were modified several times and video-conferencing options were also available. Initial meetings took place during the week at midday in a reserved classroom at the academic health science center with parking costs covered by the project team. While most SAC members attended every meeting held at this location, it was often challenging for community members to commute to campus, particularly due to its location in a medical center. The group agreed to change locations to the home offices of the local Alzheimer’s Association chapter, where a large conference room equipped with technology for slideshow presentations was available. Meetings at the new location were held during lunch hour with lunch and/or refreshments typically provided by the project team during meetings. The clinician members of the SAC were challenged by this change as the commute was difficult to accommodate in their schedules. However, representation of at least one clinician was maintained at every SAC meeting and the decision to prioritize the needs of community stakeholders was determined to be most important to ensure their representation and comfort at all meetings.

*“…but obviously [meeting] location was forever North. That’s a problem.”* – Family care partner 2

*“I wish I could be much more engaged than I am…… When they moved the meetings to Mondays, it became very difficult because Monday is my clinic day, and I couldn’t participate as much.”*- Health and Social care professional 2

Following COVID-19, all SAC meetings took place online via Zoom video conferencing software. SAC members felt that while meeting in-person was preferred, there were some benefits to attending meetings online. In particular, clinician members of the SAC were better able to attend virtual meetings in-between their clinic duties and persons living with dementia and care partners were no longer needed to travel. Attendance was maintained consistently by most members of the SAC during the virtual meetings, though there were some challenges. At times, some members were challenged with unstable internet connections, often needing to opt for telephone connection with no video, though this did not seem to fully deter their participation in discussions. Additionally, engagement with individuals living with dementia was impacted and new approaches were necessary to encourage their participation at times. Care partners and other SAC members addressed this challenge by directly addressing each other by name to invite all members to have an opportunity to share their thoughts and perspectives throughout the discussion.

*“I would say the biggest challenge is time... but for a caregiver or a member from the community or whatever, wow. That’s hard to go to [the university] and what floor and what classroom.”* – Health and social care professional 5

*“Yes, when COVID started, we had Zoom, and that platform has been used for many of our––well, it's almost our day to day life.”* – Family care partner 3

*“You start off [on Zoom] and it’s just like talking to you right now. It's a pleasure. It's enjoyable.”* - Person living with dementia 1

### Council infrastructure

SAC members expressed that the council benefitted from the diverse representation of professional and lived experiences among the group. They felt they brought something unique to the group from their different professional and lived experiences. In establishing the SAC, members were invited to the project primarily based on their professional and/or lived experiences. The project team sought to invite representatives of the stakeholder groups listed in Table 1. SAC members stressed the value of having a council representing diverse perspectives but emphasized the need to hear from persons living with dementia in particular.

*“I feel such a mutual respect for one another, personally and for their opinions, that they have value. That’s not always seen in a mixed group, as we have, I don’t think. I don’t think it’s as common as what I’ve seen with our committee group. I really appreciate it…”* – Family care partner 1

“*Well, personal opinion is if you've got somebody in the group such as me that's got the disease, I think it means more to other people. Here is somebody who has the disease. What do they have to say about it?*” – Person living with dementia 1

*“What I liked about being a part of the group was hearing from the patient and caregiver perspective what really matters to them and how, as healthcare providers, we don’t even take into consideration some of the things that would actually make a better difference in their quality of life.”* – Health and social care professional 2

Respondents felt that members of the project team responsible for facilitating the SAC activities need to be ‘dementia aware’, including having knowledge about dementia and being respectful, patient, and considerate of the accommodations individuals living with dementia and family care partners may need to stay involved. In discussing preferences for dementia aware project leads, respondents shared their conceptions of how they perceived typical researchers’ approach to engagement which was often viewed as predatory, where researchers enter communities to extract information without giving back. The SAC valued when facilitators would take a transparent approach to research, sharing their intentions in understandable terms and inviting collaboration of stakeholders throughout the research process.

*“[The facilitator’s] leadership style is intentional, it’s purposeful, it’s inclusive, and it’s also very humbling, which—I don’t think it’s a common word choice to use in research, but their research approach is very—confident enough to humble and be, like, ‘These are my areas of expertise. This area or this community is not.’… They seek it, they welcome it…”* Family care partner 2

*“[The project team] would come over before COVID and speak to [my spouse], and they respected his opinion. They treated him as a thinking human being who had something to contribute.”* – Family care partner 3

*“[The project team has] worked diligently and very hard on it. They've listened to what we've had to say.”* – Person living with dementia 1

*“Well, one of the things is, since it's a number of personnel working with us, we all have to try to come to an agreement, but it's awesome because leadership is open to see what works for everyone. There is no, "Let's talk behind closed doors and let them know what the change is." There's a question, always from the top, "How do you all feel about that? Does that work for you?" That's so inclusive in a positive way.”* – Health and social care professional 3

### Values and environment

SAC members shared distinctive collective values and standards for the group culture. Respondents across all representative stakeholder groups shared that they were valued and respected for their contributions. Having a unique space and role on the council was important to members, some reflecting a sense of pride and responsibility as representatives of a wider network of stakeholders.

*“It’s been a rewarding experience for me to feel like I’ve had a voice in it and, hopefully, have represented other caregivers well in my concerns and shared experience with those on the committee who haven’t been a caregiver.”* – Family care partner 1

*“I have had no problem at all in sharing what I felt we could do, or start, or explore. All the members have always, I think, looked forward to what I had to say.”* – Health and social care professional 2

*“I think they do (value opinions and input) particularly if they ask me a question, I answer it. There’s a whole group of us. Is anybody better than anybody else? I don't know.”* – Person living with dementia 1

A key value shared by respondents was the cultivation of an inclusive environment where SAC members could feel comfortable to share their opinions and perspectives. Inclusivity was often attributed to the non-hierarchical structure of meetings where all members of the SAC were invited to share and listen from their unique perspectives.

*“From the first day of the meeting it was very evident that that's why we were there, and everyone was sharing their thoughts and concerns, including [my spouse living with dementia].*" Family care partner 4

“*I have to say I’ve always felt safe. I’m a person who tries to create safety. Bringing our team together and making sure that we’re all equal, if you will. I feel like I’ve contributed to that, but I’ve also felt that, certainly*.” Health and social care professional 5

SAC members were motivated to participate in the project for different reasons, many expressed an altruistic incentive to contribute to dementia care research. They had a strong desire to help others with their work, recognizing their participation might inform the direction of future research that could benefit others. Their altruistic motivations extended from their desire to help their friends, families, as well as society at large. Being part of something bigger was important for SAC members who recognized that not everyone had the ability to participate in similar opportunities.

“*I know that with that responsibility comes the benefit directly to impact my family, myself, my community, society. I’m cognizant of that, and so I happily take it with that in mind*.” – Family care partner 2

“*I'm not gonna hide it. I'm gonna let everybody know I've got it, and if I can be of any help to anybody, I'm here.”* – Person living with dementia 1

Most SAC members identified the need for a cultural awareness in the group and in the activities of the project. They felt it was important to seek diverse cultural representation within the council structure and to foster an environment that is inclusive for different ethnic and cultural backgrounds. Some SAC members of stakeholder groups that are often excluded from collaboration for research, including care partners and individuals from ethnic and racial minorities, felt a sense of responsibility to represent and educate others about their lived experiences.

*“We need to get people who look like the people that we're trying to reach: the Hispanics, the African Americans, the minorities, the people who are not as privileged. It can't be the perception of this is a white person's cure.”* – Family care partner 3

*“It feels like [my] responsibility to educate the entire room. As much as they’re receptive and respectful, it’s also very exhausting.”* – Family care partner 1

#### Benefits of involvement

The activities of the SAC were primarily developed to facilitate capacity-building for patient-centered outcomes research. Participants, however, identified additional personal benefits beyond the purpose of research. The development of knowledge and skills through participation on the SAC was an added benefit for interviewees who felt they could translate their skills to their personal and professional lives. Health and social professionals often shared that engaging with individuals living with dementia and care partners in a collaborative setting, as opposed to clinical or service-based, provided the opportunity for them to integrate communication techniques into their work.

*“Certainly taking that out to the—my colleagues and my work, and we’ve implemented ways that I think we’re hopefully explaining better, helping people understand the disease better, and helping people understand what the diagnosis is gonna look like.”* - Health and social care professional 3

“*Yes, definitely, the biggest part, obviously, to me is I wanted to improve—to learn about ways to improve my caregiving with fact- based, research-based techniques that have been applied, that professional – that works, or doesn’t work, or semi-works, or it works in conjunction with A, B, C, for sure.*” – family care partner 2

Further, they felt their participation provided opportunities for socialization, building their communities, and the chance to share and listen to diverse perspectives and stories. The element of peer support and social connection was particularly resonant in the context of the COVID-19 pandemic, where SAC members were experiencing the social consequences of distancing safety precautions.

*“I don't want to use the word “fun” but fulfilling. It's been fun. It's been supportive for me, and I believe for Michael… Just knowing that we're not isolated has been extremely helpful.”* – Family care partner 3

*“[The meetings] gave some meaningful activities and something meaningful, too, for the council members. It was more like a family environment. People could just be themselves…”* – Health and social care professional 2

Another key benefit reflected in respondent interviews was that SAC members see the value in the work being done. SAC members expressed that they felt they met and exceeded the goals of the original project and had confidence that their work would make a difference in the field of dementia care research. Interviews reflected a sense of accomplishment and belief in the purpose of the project.

*"I think we accomplished a lot in that, and painstakingly, I know, for those who have been at the helm. I think a lot of useful information will be obtained for research purposes..."* – Family caregiver 1

*“I think the council has done, as I told you, much more than just focusing on the proposal. It’s been many more benefits to everybody… You do research, and you think you know it all, and you go into the community, and you ask questions, but here you are realizing that maybe what you were asking or what you were trying to research is not really what needs to be done.”* – Health and social care professional 2

## Conclusions

Engaging stakeholders in dementia care research is a national priority, yet, evaluation of engagement strategies and their impact on the experiences of stakeholders is limited [31]. This paper describes the perspectives around engagement and building capacity for research from members of a multiple stakeholder advisory council established to identify dementia care research priorities. As such, the results reported here contribute to our understanding of possible outcomes for stakeholders from their engagement in research [28]. The findings outlined in this paper are a resource for researchers seeking to collaborate with stakeholders in dementia care, to represent their voices and perspectives in research.

Although community advisory boards are an established practice for research across many disciplines, the inclusion of persons living with dementia in these spaces has been minimal [17, 32]. The limited engagement of individuals living with dementia as co-researchers may be attributed to logistical challenges related to symptoms of the condition, social barriers that limit their ability to participate, or potential to overburden or distress participants [26, 33]. Their participation in the development of meaningful research is critical, however, and efforts to encourage their engagement as collaborators are needed [28, 31]. One approach to include persons living with dementia as collaborators in research is through a multiple stakeholder advisory council. The multiple stakeholder approach can enable persons living with dementia to participate in a supportive group culture with shared project goals where they are given agency to engage in research activities without overburdening them as the primary drivers of the research process.

Domains and themes from this qualitative assessment of stakeholder engagement reflect that persons living with dementia can be supported as collaborators in research while also representing the perspectives and insights of other stakeholder groups. The themes reflected in interviews with SAC members suggest that when centering activities around the needs of persons living with dementia, other stakeholders on the council can also effectively participate and benefit from the process. This is evident across domains where a predominant viewpoint is shared among SAC members that although all members share equal footing on the council, there should be concerted effort to uplift and support those living with dementia to share their valuable insight. Council members representing diverse stakeholder groups tied the value of the project to the inclusion of persons living with dementia on the council, recognizing that this is a limited practice with the potential for significant impact on future research and service in dementia care.

Discussion around the infrastructure of the council was consistent among respondents, reflecting the importance of diverse representation while maintaining an inclusive culture where all members feel comfortable to share their insights. In building a council infrastructure and culture that is conducive to identifying and accomplishing shared goals, members needed to feel appreciation and acknowledgment for their individual roles in the group. Respondents reflected that inclusion in the research process cultivated a sense of being valued and respected in the research environment and process. However, there are challenges in developing a shared culture among a mixed group of stakeholders which can impact the productivity and engagement among the group [34]. Fostering the rapport needed to collaborate with the multi-stakeholder council took time and the project team found it necessary to regularly adapt their approach to support the development of a group identity. In line with other research efforts involving stakeholder advisory councils, there was an ongoing process of balancing the need to build rapport and a sense of community among the SAC while continuing to meet the project goals and deliverables [17].

To address the potential challenges of building a shared group identity among council members that contributes to meeting project objectives, researchers should be thoughtful about who to include and how to best represent the wider community in their group [7, 8]. Consistent with best practice as identified by Bethel et al., the intake process for invited members should involve a clear and understandable explanation of the project and its goals, as well as the importance of representation on the council as a whole and at each meeting [17]. Further, integrating activities that foster closer relationships among the group in conjunction with research capacity building activities should be considered at the onset of the project.

Findings suggest the potential for multi-stakeholder advisory councils to inform dementia care research by centering engagement strategies around individuals living with dementia. Despite the unique challenges associated with involving persons living with dementia and family care partners as co-researchers, their voices are critical to ensure the relevance of research in dementia care. Including them in an inclusive collaborative research environment adapted to their needs may support their engagement in the research process. To sustain engagement among an advisory council for research, adapting as needed is key and allows the project to define itself while still meeting the prescribed research activities and deliverables. Researchers should not underestimate the value in going at the pace of their unique group of council members, which ultimately results in stronger rapport and more productive meetings over time [29]. Taking a strengths-based approach, where all members are acknowledged and supported for the unique experiences they bring to the project can contribute to productivity while fostering a sense of purpose in each contributing member.

In assessing engagement among a diverse stakeholder advisory council for dementia care research, this evaluation adds to limited knowledge of the potential for including persons living with dementia as co-researchers. Further, key themes from this evaluation can guide researchers seeking to include stakeholders in the research process and can serve as a framework for their engagement efforts (see table of key learnings in supplemental materials). This study is limited by the small sample of persons living with dementia and family care partners, attributed to the resignation of some participants from the project related to progression of the dementia. From our experience working with the SAC and the loss of representation among individuals living with dementia we experienced over time, we recommend increasing representation in numbers among this stakeholder group at the initial recruitment stage and encouraging new membership through the project period. We also recommend being clear of expectations at the time of recruitment, outlining the project timeline, functions of the SAC, individual expectations, and the projected meeting frequency and structure. This creates an opportunity to better support individuals living with dementia at the project onset and may sustain their engagement throughout the project period. We were also limited in our representation of caregivers and individuals living with dementia who identify as ethnic and racial minorities. While diversity for this project was primarily represented in roles as professionals in dementia care and lived experiences as caregivers or individuals living with dementia, only a few community members of the SAC identified as ethnic or racial minorities. There is a need for intentionally focused recruitment of community and stakeholder advisory councils for dementia care research engagement that represent minority groups who are at increased risk for dementia [35]. Further, the COVID-19 pandemic posed challenges related to conducting the interviews, particularly for persons living with dementia. To address this, every accommodation that could be offered within safety parameters was offered, including video conferencing, telephone interviews, the option to complete interviews in multiple sessions, and the choice to have a care partner present if preferred.

Meaningful engagement of stakeholders comes with challenges as noted here, with the results also reflecting the importance of the principles of transparency, inclusivity, and trust, guided by the Scottish experience [29], that we established early in the process. The model of engagement reported here has shaped our entire project from inception through to achieving our goal of a prioritized list of topics for dementia care research [15] and research examining the impact of COVID-19 on dementia care, a priority of the SAC. The process of engagement and the results reported here add to the evidence base about stakeholder collaboration in research and can inform researchers who are seeking guidance in including stakeholders in their program of research. There is a need for research that examines the impact of engagement on dissemination and implementation of research findings to ultimately impact the care of families for which the research was intended.

## Supplementary Information

**Additional file 1: Table 2.** GRIPP2 short form.

**Additional file 2: Supplemental Table 1.** Recommendations based on key learnings from engagement with a Stakeholder Advisory Council for PCOR in dementia care.

## Data Availability

The datasets used and/or analyzed during the current study are available from the corresponding author on request.

## Abbreviations

PCORI: Patient Centered Outcomes Research Institute
CER: Community Engaged Research
SAC: Stakeholder Advisory Council
COVID-19: Coronavirus Disease 2019
IRB: Institutional Review Board
