# Supplementary material for: Engagement with a diverse Stakeholder Advisory Council for research in dementia care
Source: Res Involv Engagem. 2021 Jul 23;7:54. doi: 10.1186/s40900-021-00297-8 (PMC8300992; doi:10.1186/s40900-021-00297-8)
Supplement: Supplementary file 1 — Additional file 1: Table 2. GRIPP2 short form. [file 40900_2021_297_MOESM1_ESM.docx]

GRIPP2 Short Form

**Table 2** GRIPP2 short form

| Section and topic | Item | Reported on page No |
| --- | --- | --- |
| 1: Aim | Report the aim of PPI in the study | 9 |
| 2: Methods | Provide a clear description of the methods used for PPI in the study | 9-13 |
| 3: Study results | Outcomes—Report the results of PPI in the study, including both positive and negative outcomes | 16-27 |
| 4: Discussion and conclusions | Outcomes—Comment on the extent to which PPI influenced the study overall. Describe positive and negative effects | 27-32 |
| 5: Reflections/critical perspective | Comment critically on the study, reflecting on the things that went well and those that did not, so others can learn from this experience | 30-32 |

PPI=patient and public involvement
