# Supplementary material for: Engagement with a diverse Stakeholder Advisory Council for research in dementia care
Source: Res Involv Engagem. 2021 Jul 23;7:54. doi: 10.1186/s40900-021-00297-8 (PMC8300992; doi:10.1186/s40900-021-00297-8)
Supplement: Supplementary file 2 — Additional file 2: Supplemental Table 1. Recommendations based on key learnings from engagement with a Stakeholder Advisory Council for PCOR in dementia care. [file 40900_2021_297_MOESM2_ESM.docx]

| **Supplemental Table 1. Recommendations based on key learnings from engagement with a Stakeholder Advisory Council for PCOR in dementia care** | |
| --- | --- |
| **Domains** | **Recommendations** |
| **Accessibility** | - Use a common language to communicate and translate research in an accessible and inclusive way (i.e., avoid acronyms). Guidelines for the group to support accessible language can be determined through semi-structured discussions in initial meetings that can be revisited as needed over time and with new members. - Make use of a variety of formats and mediums for information sharing to support all members to participate in activities and discussions about research (e.g., visual alternatives and large print fonts). Plan to revisit important topics over time to reinforce key areas of capacity building for research. - As a group, agree on meeting times and locations to support all members of the council, prioritizing the needs of persons living with dementia and family care partners. |
| **Council infrastructure** | - In establishing your council, invite multiple members representing each of the key stakeholder groups you identify as important to your project. Ask members who else is “not at the table” and should be included in the council. - Prioritize cultural and ethnic representation on the council within stakeholder groups. - Aim to prioritize greater representation of primary stakeholder groups (i.e., individuals living with dementia) to promote diversity in experiences and perspectives within the sub-group. - As a council, decide on a structure and approach to collaboration with a goal to promote equal footing among members, limiting the chance for some to dominate discourse over others. - Project team and council members should have experience and knowledge about dementia and be respectful, patient, and considerate of the needs of individuals living with dementia and family care partners. |
| **Values & environment** | - Aim to cultivate an environment where members feel their contributions are respected and valued by others on the council and the project team (i.e., decisions made by the council are supported and implemented by the project team). - Project team facilitate an environment where council members feel comfortable to share their opinions with the group. Discuss this with the council to understand the barriers and facilitators to their sharing. - Whenever possible, compensate council members fairly for their time at meetings and other activities. - Discuss and revisit as needed how the work being done by the council can support other community members and contribute to meaningful change. - Prioritize respectful discussions of cultural and ethnic representation and awareness among the group. Address |
| **Benefits of involvement** | - Make room in meetings for members to share their personal motivations for participating on the council and contributing to research. - Celebrate council accomplishments during the project period, acknowledging the unique roles individual members play in successfully meeting goals and objectives. - Report back project outcomes and deliverables completed during and after the project period to the council and wider community. Develop resources and updates for members to share with their families, friends, and networks. |
